# The phase average wavelength of alpha rhythm in EEG signals of patients with Parkinson’s disease combined with cognitive impairment

**DOI:** 10.1371/journal.pone.0344786

**Published:** 2026-04-22

**Authors:** Lifang Zhao, Beibei Zhang, Ying Zhang, Nan Jiang, Yan Wei, Lijun Hao, Meiyun Zhang

**Affiliations:** 1 Tianjin Union Medical Center, Tianjin Medical University, Tianjin, China; 2 Department of Neurology, Zhongda Hospital Southeast University, Nanjing, Jiangsu, China; 3 Department of Neurology, Tianjin Union Medical Center, The First Affiliated Hospital of Nankai University, Tianjin, China; 4 School of Mechanical Engineering, Tianjin University, Tianjin, China; Utano National Hospital, JAPAN

## Abstract

**Objective:**

To investigate the changes of phase average wavelength of alpha rhythm in different brain regions of patients with Parkinson’s Disease (PD) during awake period with eyes closed.

**Methods:**

This study included 54 patients with PD and 30 normal controls. The PD patients were divided into a PD-cognitive normal group (PD-NC) and a PD-cognitive impairment group (PD-CI) based on Mini-Mental State Examination (MMSE) scores. The electroencephalography signals were decomposed at different scales using the Gauss continuous wavelet transform method. The phase average wavelength of the ninth scale (corresponding to the alpha rhythm) was extracted using the conditional sampling and phase averaging method. The alpha-band wavelengths of the background rhythm of different leads were compared between groups. Additionally, the correlation between the wavelength of the ninth scale and MMSE and Montreal Cognitive Assessment (MoCA) scores in PD patients was analyzed.

**Results:**

There were significant differences in phase average wavelength at scale nine in each lead among the three groups (*P* < 0.05). The phase average wavelength of the alpha rhythm in all brain regions corresponding to the ninth scale in PD patients was prolonged compared with the normal controls (*P* < 0.05). There was a tendency for the PD-CI group to be longer than the PD-NC group, especially in the frontal area, central area and anterior temporal area. The average wavelength of alpha rhythm phase was negatively correlated with MoCA score in PD patients in all leads (*P* < 0.05), except the right central region (C4) and middle temporal region (T4).

**Conclusions:**

The phase average wavelength of the alpha rhythm in PD patients was longer than that in normal controls, indicating that the alpha rhythm of PD patients was slowed down. With the deterioration of cognitive function, the alpha rhythm of PD patients gradually slowed down.

**Significance:**

The phase average wavelength of the electroencephalography alpha rhythm may become a new parameter for the evaluation of cognitive impairment.

## 1. Introduction

Parkinson’s disease (PD) [1] is the second most common neurodegenerative disorder, primarily characterized by motor symptoms including resting tremor, bradykinesia, rigidity, and postural instability. However, growing evidence suggests that PD is not merely a motor disorder but also presents with a range of non-motor symptoms, among which cognitive impairment is a particularly common and significant manifestation [2–4]. Severe cognitive impairment in PD, known as Parkinson’s disease dementia (PDD), typically manifests in advanced stages of the disease [5]. Foltynie et al [6] indicates that cognitive impairment can emerge even in the prodromal phase of PD, significantly impairing patients’ quality of life. Due to insufficient recognition of non-motor symptoms, mild cognitive impairment (MCI) is often overlooked by patients, their families, and even physicians. A meta-analysis [7] reported that over a three-year follow-up period, among initially cognitively normal PD patients, 25% progressed to PD-associated mild cognitive impairment (PD-MCI) and 2% to PDD. Among patients with PD-MCI at baseline, 20% progressed to PDD during the same period. This meta-analysis further highlighted that conversion rates to both MCI and dementia are notably high with follow-up periods of ≥3 years. In a 20-year follow-up study of newly diagnosed PD patients, Hely et al [8] found that among survivors at 20 years, a striking 83% had developed PDD. They also noted a correlation between dementia onset and advancing age. Furthermore, post-mortem examinations in some patients revealed widespread Lewy body pathology, leading them to hypothesize that PD-related cognitive impairment may result from the interplay of multiple pathological factors.

The motor symptoms of PD are primarily attributed to the loss of dopaminergic neurons in the substantia nigra. Early clinical symptoms are often difficult to recognize until substantial dopaminergic neuronal damage occurs, leading to more prominent clinical manifestations. Beyond the dopaminergic system, PD patients also exhibit dysfunction in other neurotransmitter systems, including cholinergic, noradrenergic, and serotonergic pathways, all of which contribute to cognitive regulation [9–13]. Accumulating evidence strongly suggests that alterations in the cholinergic system are closely linked to cognitive decline in PD patients [14]. Schumacher et al [15] conducted in vivo studies employing structural MRI to assess basal forebrain volume and PET to measure cortical cholinergic activity. Their findings revealed that basal forebrain degeneration in PD is accompanied by changes in cortical acetylcholinesterase activity. Moreover, cholinergic imaging markers detected by both PET and MRI demonstrated an independent association with multi-domain cognitive deficits in non-demented PD patients. In contrast, hippocampal atrophy appears to have only a weak association with the progression of early cognitive impairment in PD.

Cognitive impairment associated with PD can be broadly classified into two main profiles. The first primarily manifests as deficits in planning, working memory, and executive functions linked to fronto-striatal circuit dysfunction, often accompanied by dopaminergic hypofunction. The second profile is predominantly characterized by impairments in attention, language, and visuospatial abilities, with its neuropathological underpinnings involving not only temporal lobe but also occipital lobe cortical dysfunction [16,17]. Some PD patients present with cognitive impairment at the time of diagnosis, and in most individuals, this cognitive dysfunction follows a progressive course [18]. Early identification and intervention for patients at high risk of PD-related cognitive impairment can help slow the progression of cognitive decline, thereby improving their quality of life [19]. Consequently, from the perspectives of both enhancing patient prognosis and developing effective therapeutic strategies, the early diagnosis and assessment of PD-associated cognitive impairment are of significant clinical and societal importance. The assessment of PD-associated cognitive impairment currently relies heavily on standardized neuropsychological scales, such as the Mini-Mental State Examination (MMSE), the Montreal Cognitive Assessment (MoCA), and pertinent sections of the Unified Parkinson’s Disease Rating Scale (UPDRS). A notable limitation of these scale-based evaluations, however, is their susceptibility to subjective influences, including inter-rater variability and patient compliance. In recent years, electroencephalography (EEG), a non-invasive neuroelectrophysiological technique characterized by its high temporal resolution, has gained increasing prominence in the investigation of cognitive impairment in PD. Quantitative electroencephalography methodologies facilitate the extraction of diverse parameters from EEG signals, encompassing frequency content, power spectral density, and functional connectivity metrics. When integrated with neuropsychological test performance, these objective quantitative electroencephalography markers offer the potential to furnish a more robust and reliable basis for evaluating cognitive decline.

Quantitative electroencephalography demonstrates broad utility in clinical research on PD, encompassing the assessment of cognitive function, monitoring of therapeutic responses, and investigation into the neural mechanisms of motor impairments. Caviness et al [20] stratified PD patients by cognitive status into cognitively normal (PD-CogNL), mild cognitive impairment (PD-MCI), and dementia (PDD) groups, subsequently analyzing their resting-state EEG rhythms and power across five principal frequency bands. Similarly, another study observed a comparable trend of EEG slowing in PD patients with increasing motor severity [21]. The motor symptoms of PD are considered to be closely associated with aberrant neural oscillations within the basal ganglia-thalamocortical circuitry [22]. Analysis of the phase dynamics of neural oscillations during distinct stages of movement, such as motor preparation and execution, in PD patients has elucidated aspects of the neural underpinnings of motor dysfunction [23–25]. Given that cognitive impairment is a frequent comorbidity in PD, and cognitive functions are known to be intricately linked to the time- domain and frequency-domain characteristics of neural oscillations [24]. Quantitative electroencephalography presents a valuable methodology for investigating cognitive deficits in this patient population.

The Phase Average Waveform (PAW) is an analysis method based on the phase and amplitude information of EEG signals, capable of reflecting the time-domain characteristics of neural oscillations. PAW first uses the continuous wavelet transform method to decompose the EEG signal into different scales; a conditional function is set, signals meeting the detection criteria at a certain scale are retained, otherwise they are removed. The retained EEG signals are superimposed in a phase-aligned manner and then averaged to obtain the phase-averaged waveform for that scale. The advantages of the PAW are, on one hand, that it embodies time-domain features such as phase, amplitude, and wavelength, and on the other hand, the process of decomposition through wavelet transform excludes interference from signals of other scales. The wavelength of PAW reflects the frequency characteristics of the EEG signal at a specific scale.

PAW was first applied in turbulence research [26], and later applied to the study of EEG signals in Alzheimer’s disease(AD) [27]. Currently, there is still a lack of systematic research on PAW in the EEG signal analysis of PD patients with cognitive impairment. This study aims to analyze the phase average wavelength characteristics of PD patients with cognitive impairment and evaluate the correlation between quantitative parameters extracted therefrom and clinical cognitive function scale scores.

## 2. Materials and methods

### 2.1 Participants

A total of 54 PD patients (29 males and 25 females) were included in this study, mainly from the outpatient and inpatient departments of neurology, Tianjin Union Medical Center, between November, 2021 and September, 2024. Thirty normal controls were enrolled. PD patients were divided into two groups according to MMSE score. MMSE score ≥26 was divided into PD cognitive normal group (PD-NC), with a total of 30 cases. A total of 24 patients with MMSE<26 was divided into PD cognitive impairment group (PD-CI).

PD group inclusion criteria: (1) age 45–85 years old; (2) According to the 2015 International Parkinson and Movement Disorder Society (MDS) clinical diagnostic criteria for Parkinson’s disease [28]; (3) the neuropsychological function test can cooperate to complete.

Exclusion criteria: (1) secondary Parkinson’s disease and Parkinson’s plus syndrome; (2) history of severe organic chronic diseases of the heart, brain, lung, liver, or kidney; (3) severe cognitive impairment, dementia and mental abnormalities were unable to cooperate with the completion of the scale and EEG signal collection.

The main symptoms, onset years, and education level of PD patients were collected. The motor symptoms and non-motor symptoms (cognitive function, sleep, and depression) of PD patients were assessed by neurological scales. Cognitive function was assessed by MMSE. The cut-off value below 26 was defined as cognitive impairment, and the cut-off value above 26 was defined as normal. MoCA scale was used in all PD patients. If the education level was ≤ 12 years, 1 point was added, and the maximum score was 30. The Unified Parkinson’s Disease Rating Scale (UPDRS Ⅱ-Ⅲ) is used to assess the severity of daily activities and motor symptoms. The higher the score, the more severe the symptoms. The Hoehn and Yahr (HY) scale (stages 1–2 are mild, stages 2.5–3 are moderate, and stages 4–5 are severe) assesses the staging of motor symptom severity. Hamilton Depression Scale-17item (HAMD-17) score was used to evaluate depressive symptoms, the total score <7: normal; Total score between 7 and 17: possible depression: total score between 17 and 24: definite depression. Pittsburgh sleep quality index (PSQI) was used to evaluate the sleep status of PD patients.

This study was approved by the Ethics Committee of Tianjin Union Medical Center, The First Affiliated Hospital of Nankai University (Approval number: 2021-B24). Written consent was obtained from all patients.

### 2.2 Research methods

#### 2.2.1 EEG signal acquisition.

All participants had their EEG data collected using the EB-neuro Be-light (Firenze, Italy) EEG recording system. Electrodes were placed according to the international 10/20 system, with both ears as the reference electrodes. Sixteen-channel EEG signals were collected, with the leads set as follows: FP1-A1, FP2-A2; F3-A1, F4-A2; C3-A1, C4-A2; P3-A1, P4-A2; O1-A1, O2-A2; F7-A1, F8-A2; T3-A1, T4-A2; T5-A1, T6-A2. The sampling frequency was 512 Hz, with an impedance of less than 5KΩ. The low-frequency and high-frequency filters were set at 0.3 Hz and 30 Hz, respectively. After collecting the EEG data, 20s raw EEG without visual artifacts was selected for quantitative analysis of the 16-lead EEG data. All subjects completed EEG data acquisition in a quiet, well-soundproof EEG room, and recorded EEG signals in the sitting position, awake, quiet and closed eyes for at least 20 minutes. On the day before the examination, the subjects were reminded to wash their scalp, have adequate rest, prohibit stimulants (such as tea, alcohol, coffee, etc.) and drugs (such as antipsychotic drugs, anti-anxiety and depression drugs), and maintain good mental state without fever and other symptoms on the day of the record. Parkinson’s drugs were routinely used before and after the examination. All patients’ condition information, EEG electrode placement and EEG signal acquisition were completed by neurology specialists.

#### 2.2.2 Gauss continuous wavelet analysis.

The Gauss continuous wavelet transform method was used to analyze the multi-scale EEG data. EEG data is a non-stationary complex signal, and wavelet analysis through Continuous Wavelet Transform (CWT) has good resolution ability for non-stationary signals, which is suitable for EEG signal processing. The formula is as follows:


$$\begin{array}{c} CWTf(\alpha ,\beta )=\int_{-\infty }^{+\infty }f(t)\cdot \psi_{\alpha ,\beta }(t)dt \end{array}$$ (1)

The $\psi_{\alpha ,\beta }(t)$ wavelet function set is obtained by translating the wavelet parent function by β units and scaling it by α times.

**Fig 1:**
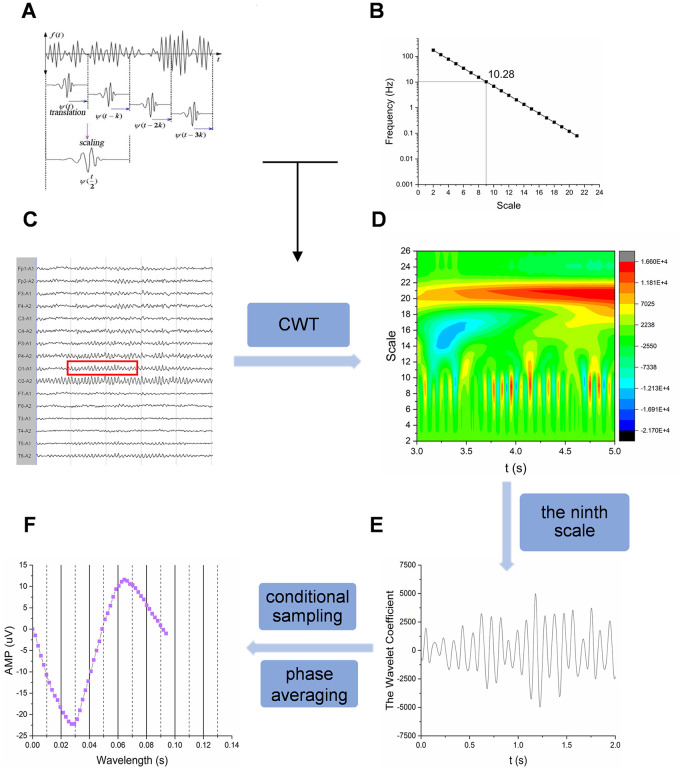
EEG analysis flowchart. (A) Signal decomposition and extraction process of the wavelet transform signal. (B) Correspondence between scale and frequency. (C) Original visual EEG. (D) Time-frequency analysis of EEG signals at different scales. (E) The waveform activity of the EEG signal in the occipital region lasting for 2 seconds at the ninth scale. (F) The phase average waveform wavelength of lead O1.


Where t represents time, α represents the scaling factor, and β represents the translation parameter corresponding to the time variable t. The translation and scaling process is shown in Fig 1A. The formula for the wavelet function is as follows:

$$\begin{array}{c} \psi_{\alpha ,\beta }(t)=\frac{\text{1}}{\sqrt{\alpha }}\psi \left(\frac{t-\beta }{\alpha }\right) \end{array}$$ (2)

After CWT, the original EEG signal is decomposed in different scales, which are divided into 1–30 scales in total. The wavelet function scale number and its corresponding EEG signal frequency correspondence are shown in Fig 1B.

#### 2.2.3 Conditional sampling-phase averaging.

The extraction of an α-scale phase average waveform takes the single wavelet coefficient W(t,α) of continuous EEG signal f(t) at this scale as the detection condition, and the detection condition is that W(t,α) takes its maximum positive value. The detection function is defined as follows:


$$\begin{array}{c} @lD(t,\alpha )=\left\{ \begin{array}{c} @l\text{1},t\in \left[t_{i}-\frac{T(\alpha )}{\text{2}},t_{i}+\frac{T(\alpha )}{\text{2}}\right]\\ \text{0},otherwise \end{array}\right.W(t,\alpha )\geq \text{0},W(t,\alpha )\text{is the local maximum} \end{array}$$ (3)

T(α) is the wavelength of the wavelet coefficient of scale α. This formula limits the sampling function under certain conditions, also known as conditional sampling function. The above equation (3) is multiplied by the original EEG signal f(t) to obtain f(t)D·(t,α)*.* Then the conditional sampling of the original continuous and complex EEG signal can be realized, the test signals that meet the conditions are retained, and the signals that do not meet the conditions are removed. This is the conditional sampling of EEG signals. In this way, the EEG signal is truncated into signal segments with the same wavelength, amplitude and phase according to a standard. Several segments of the extracted EEG signals were aligned according to phase, and then superimposed and averaged to obtain the PAW at this scale.


$$\begin{array}{c} <f(t,\alpha )\geq \frac{\text{1}}{N}\sum_{i=\text{1}}^{N}f\left(t+t_{i}-\frac{T(\alpha )}{\text{2}}\right)D\left(t_{i},\alpha \right),t\in \left[-\frac{T(\alpha )}{\text{2}},\frac{T(\alpha )}{\text{2}}\right] \end{array}$$ (4)

N represents the number of detected EEG signal fragments.

The analysis process of EEG signals is shown in Fig 1. Taking the O1 lead in EEG of normal controls as an example, the original EEG signals are decomposed by Gauss CWT, so as to study the time-frequency characteristics of EEG signals and understand the relationship between EEG signals of different scales. Fig 1D shows the time-frequency analysis diagram of the corresponding occipital region EEG signal. The horizontal axis represents time (sampling frequency 512 Hz, corresponding to 1s when converted to time), and the vertical axis represents scale (the corresponding relationship with frequency is shown in Fig 1B. Different colors represent the amplitudes (voltages) of the wavelet coefficients. Fig 1E shows the waveform activity of the EEG signal in the occipital region at the ninth scale (corresponding to the frequency center of 10.28 Hz, corresponding to the α frequency band) for 2 seconds, and the wavelength of the phase average waveform of the ninth scale is extracted (Fig 1F).

### 2.3 Statistical analysis methods

SPSS.26.0 software was used for statistical analysis. The measurement data were expressed as mean ± standard deviation. The t-test is used to compare the mean values of normally distributed data among samples. Categorical data are presented as numbers. One-way analysis of variance was used to compare the means of multiple normally distributed samples and multiple comparisons of the sample means, and Levene’s test was used for homogeneity of variance. The least significant difference (LSD) method was used for multiple comparisons when the variances were homogeneous. When variances were uneven, multiple comparisons were performed using the Tamhane T2 method. For correlation analysis, Pearson linear correlation analysis was used if the two variables followed normal distribution, and Spearman correlation analysis was used if the variance was not equal. Categorical variables were compared by chi-square test. P < 0.05 indicated statistical significance.

## 3. Results

### 3.1 Clinical data

The comparison results of the three groups of clinical data collected are shown in Table 1. There were no significant differences in gender, age, duration of disease, years of education, H-Y stage, HAMD depression score and PSQI score between the two PD groups (*P* > 0.05). There were significant differences in UPDRS motor scores Ⅱ and Ⅲ, MMSE and MoCA (*P* < 0.05). There was no significant difference in age and gender among the three groups (*P* > 0.05).

**Table 1:** Demographic and clinical characteristics of participants in the three group.

	PD		NC	t/F value	P value
	PD-NC	PD-CI			
Sex (M/F)	15/15	14/10	16/14	0.373^a^	0.830
Age (years)	67.53 ± 8.11	70.25 ± 7.21	65.20 ± 7.237	2.983	0.056
Disease duration (years)	4.31 ± 4.33	5.72 ± 3.65	—	−1.276	0.208
Education (years)	11.13 ± 2.68	9.33 ± 3.92	—	1.998	0.051
UPDRS Ⅱ	12.30 ± 6.93	17.70 ± 7.32	—	−2.780	0.008*
UPDRS Ⅲ	15.53 ± 7.90	21.46 ± 7.83	—	−2.752	0.008*
H-Y stage	2.10 ± 0.98	2.30 ± 1.30	—	−0.620	0.538
MMSE score	27.57 ± 1.30	21.08 ± 3.74	—	8.861	0*
MOCA score	21.97 ± 3.60	15.79 ± 4.35	—	5.710	0*
HAMD score	8.80 ± 5.93	11.54 ± 5.74	—	−1.713	0.093
PSQI score	9.27 ± 4.02	11.75 ± 5.23	—	−1.975	0.054

**Notes:** ^a^ Represents χ^2^ value; *Represents *P*  <  0.05.

**Abbreviations**: PD-NC, PD with normal cognition; PD-CI, PD with cognitive impairment; NC, normal control group.

### 3.2 Computer diagram in quiet and awake state with eyes closed

In a normal adult’s quiet and awake state with closed eyes, the background EEG activity is mainly the 8–13 Hz alpha rhythm, accounting for more than 80%. The occipital region is the most active, followed by the temporal and frontal regions. The EEG activity level in the parietal region is low, and the alpha rhythm is the dominant rhythm when awake and relaxed. The wavelength of the normal alpha band (10.28 Hz) is about 0.07 to 0.125 s [29].

**Fig 2:**
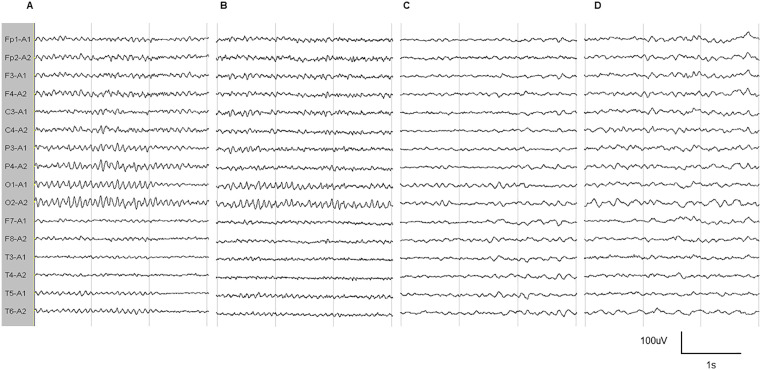
Visual EEG of the NC group and PD group subjects. (A) Normal subject (MMSE 30 points). (B) PD patient (MMSE 28 points). (C) PD patient (MMSE 20 points). (D) PD patient (MMSE 12 points).

The original EEG of 1 patient in the NC group, 1 patient in the PD-NC group, and 2 patients in the PD-CI group were selected from the data of all subjects, as shown in Fig 2. In normal subjects (MMSE 30 points) and PD patients (MMSE 28 points), EEG showed alpha rhythm (8–11 Hz) in the occipital region. In PD patients (MMSE 20), the α rhythm in occipital region was slowed down (6–8 Hz), and the α rhythm was generalized. In PD patients (MMSE 12), the α rhythm disappeared in the occipital region, and the diffuse low-medium amplitude θ rhythm was dominant.

### 3.3 Analysis of the phase average waveform at the ninth scale

#### 3.3.1 The phase average wavelength of the EEG signal at the ninth scale.

**Fig 3:**
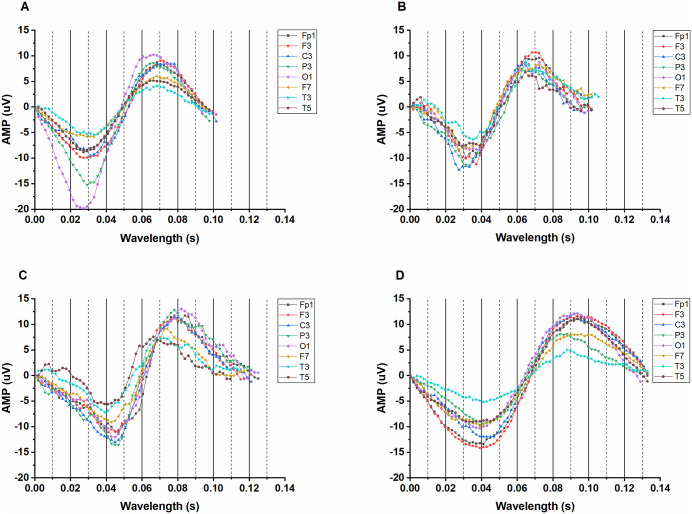
The phase average wavelength of the ninth scale in the left head region for normal control and PD subjects. (A) Normal subject (MMSE 30 points). (B) PD patient (MMSE 28 points). (C) PD patient (MMSE 20 points). (D) PD patient (MMSE 12 points).

The phase average waveforms of all leads (FP1, F3, C3, P3, O1, F7, T3, T5) on the left head of the four subjects with different scores at the ninth scale are shown in Fig 3. In the figure, Fig 3A is the normal control (MMSE = 30) with a wavelength range of about 0.1 s; Fig 3B shows a PD patient (MMSE = 28) with wavelengths ranging from 0.1 to 0.11 s in all leads of the left head. Fig 3C shows the wavelength range of all leads of the left head between 0.12 and 0.129 s in PD patients (MMSE = 20). Fig 3D shows a PD patient (MMSE = 12) with wavelength range ≥ 0.129 s in all leads of the left head. As can be seen from Fig 3, compared with the normal control group, with the decrease of MMSE score, the average wavelength of the ninth scale phase in PD patients is prolonged. The results of the right head leads (FP2, F4, C4, P4, O2, F8, T4, T6) of the four subjects obtained through the same research method are the same as those of the left head lead study.

#### 3.3.2 Comparison of the total phase average wavelengths at the ninth scale among the three groups.

To further explore the potential significance of wavelength changes in the phase average wavelength at the ninth scale in patients with PD, in this study, the wavelengths of 16 leads (FP1, F3, C3, P3, O1, F7, T3, T5, FP2, F4, C4, P4, O2, F8, T4, T6) on the ninth scale of the NC group, PD-NC group and PD-CI group were systematically analyzed.

**Fig 4:**
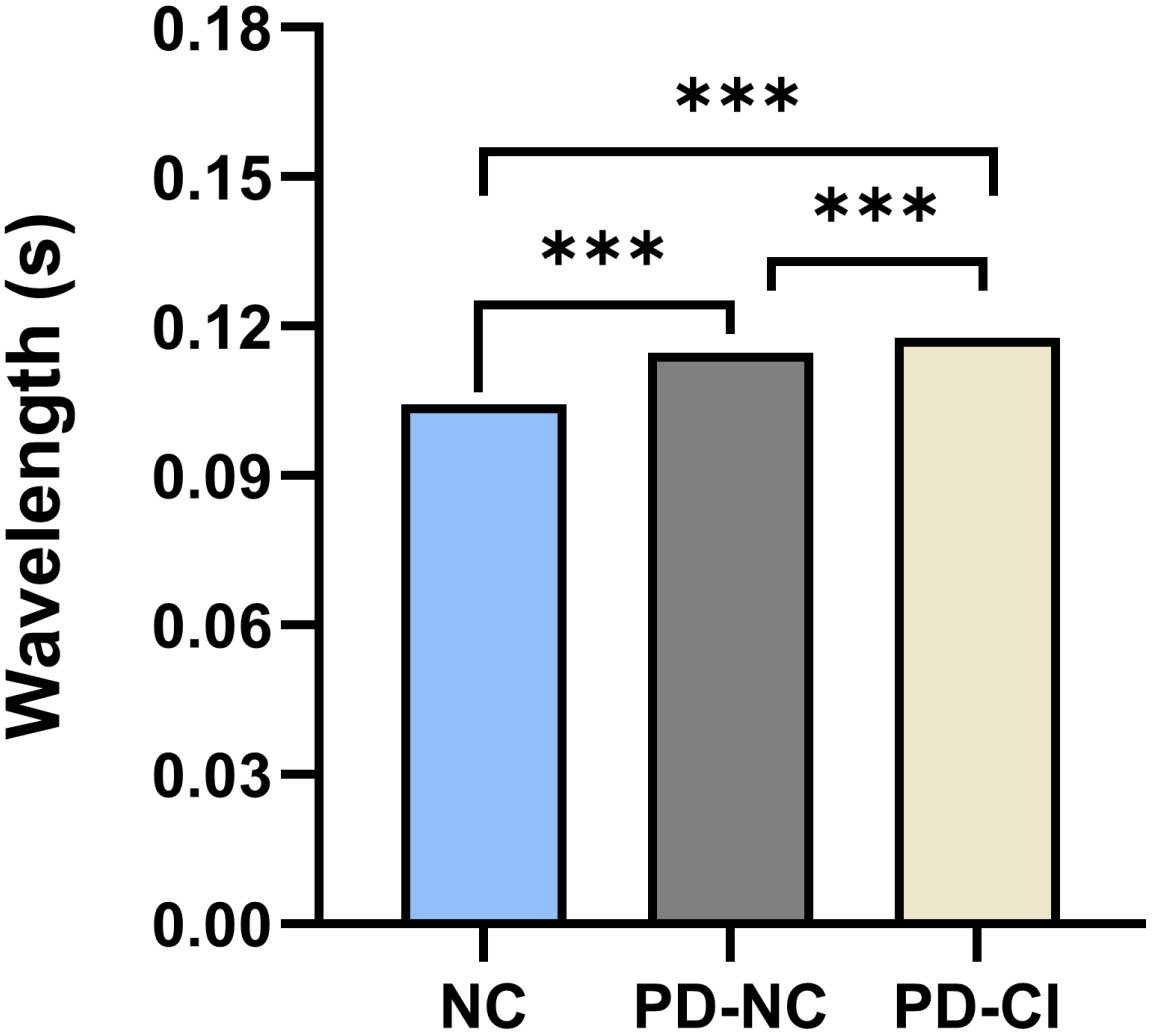
Differences in the total phase average wavelengths across the three groups. Abbreviations: PD-NC, PD with normal cognition; PD-CI, PD with cognitive impairment; NC, normal control group.

The total phase average wavelengths at the ninth scale were compared among the three groups of subjects. One-way ANOVA showed that the differences among the three groups were statistically significant (Table 2, F = 164.626, *P* < 0.001). Compared with PD-NC group and PD-CI group, the wavelength of NC group was significantly shorter. The wavelength of the PD-CI group was significantly longer than that of the PD-NC group (Fig 4, *P* < 0.001).

**Table 2:** Comparison of the total phase average wavelengths of the ninth scale across the three groups.

	Mean ± SD	F value	P value
NC	0.1043 ± 0.0090	164.626	0.000
PD-NC	0.1147 ± 0.0129		
PD-CI	0.1177 ± 0.0129		

#### 3.3.3 Comparison of the phase average wavelength across leads at the ninth scale among the three groups.

**Fig 5:**
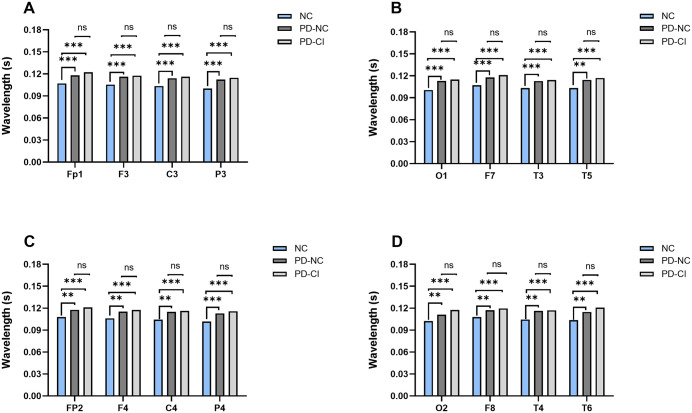
Differences in the phase average wavelength across leads of the ninth scale among three groups. Notes: ***Represents *P* < 0.001; **Represents *P* < 0.01; *Represents *P* < 0.05; ns represents *P* > 0.05. Abbreviations: PD-NC, PD with normal cognition; PD-CI, PD with cognitive impairment; NC, normal control group.

After discovering differences in the total phase average wavelengths of the three groups of subjects, further analysis of the data in 16 leads was conducted to determine the brain regions with the most significant wavelength prolongation. As shown in Table 3, data are presented as mean ± standard deviation. One-way ANOVA confirmed that all leads were significantly different among the three groups (all *P* < 0.05). Compared with the two PD subgroups (PD-NC and PD-CI), the normal control group had significantly shorter wavelengths across all 16 leads (Fig. 5, all P < 0.05). The phase average wavelength of the whole brain regions in the PD cognitive impairment group showed a prolonged trend, especially in the frontal area, central area and anterior temporal area. However, no statistically significant differences were observed between the two PD subgroups (PD-NC vs. PD-CI) for any individual lead (all *P* > 0.05). Detailed statistical results for comparisons are provided in S1 Table.

**Table 3:** Comparison of the phase average wavelength of the ninth scale leads among the three groups.

Lead	Wavelength (Mean ± SD)			F value	P value
	NC	PD-NC	PD-CI		
FP1	0.107 ± 0.0080	0.118 ± 0.0136	0.122 ± 0.0112	13.877	0.000
FP2	0.108 ± 0.0087	0.117 ± 0.0137	0.121 ± 0.0137	8.987	0.000
F3	0.105 ± 0.0069	0.116 ± 0.0118	0.118 ± 0.0105	12.768	0.000
F4	0.106 ± 0.0086	0.115 ± 0.0130	0.118 ± 0.0121	7.968	0.001
C3	0.103 ± 0.0068	0.114 ± 0.0121	0.116 ± 0.0118	12.081	0.000
C4	0.104 ± 0.0090	0.115 ± 0.0136	0.116 ± 0.0123	8.831	0.000
P3	0.100 ± 0.0070	0.112 ± 0.0113	0.115 ± 0.0126	16.189	0.000
P4	0.102 ± 0.0077	0.113 ± 0.0123	0.116 ± 0.0150	10.873	0.000
O1	0.101 ± 0.0086	0.113 ± 0.0120	0.115 ± 0.0130	14.044	0.000
O2	0.102 ± 0.0103	0.111 ± 0.0117	0.117 ± 0.0160	9.822	0.000
F7	0.107 ± 0.0081	0.118 ± 0.0131	0.121 ± 0.0120	11.860	0.000
F8	0.108 ± 0.0105	0.117 ± 0.0142	0.120 ± 0.0114	7.463	0.001
T3	0.103 ± 0.0070	0.113 ± 0.0122	0.114 ± 0.0108	9.865	0.000
T4	0.105 ± 0.0123	0.113 ± 0.0136	0.117 ± 0.0150	6.121	0.003
T5	0.103 ± 0.0101	0.114 ± 0.0137	0.117 ± 0.0130	9.903	0.000
T6	0.104 ± 0.0092	0.115 ± 0.0141	0.121 ± 0.0152	12.356	0.000

**Abbreviations:** PD-NC, PD with normal cognition; PD-CI, PD with cognitive impairment; NC, normal control group.

### 3.4 Correlation between the phase average wavelength and cognitive function scores in PD patients

**Fig 6:**
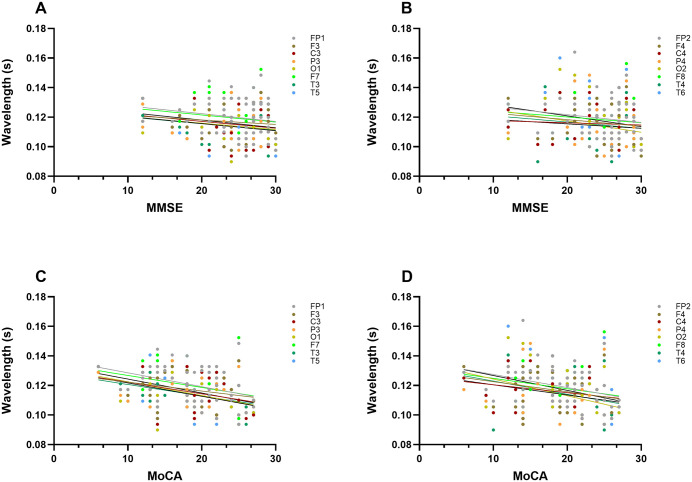
Differences in correlations between wavelengths in different brain regions and MoCA and MMSE scores.

To evaluate the trend between the phase average wavelength and cognitive function in patients with PD, and to analyze the correlation of wavelength with MoCA and MMSE scores. Table 4 presents the correlation coefficients between the two cognitive scores and the phase average wavelength in all 16 brain regions. It can be found that the association of the MoCA score with wavelength is statistically significantly greater than that of the MMSE score. The phase average wavelength and MoCA scores were negatively correlated in 13 brain regions (81.3% of the total, *P* < 0.05), and 10 of them were highly significant (*P* < 0.01), indicating that the lower the cognitive score, the longer the wavelength. The correlation between the right central region (C4, r = −0.207, *P* = 0.067, R^2^ = 0.0428) and the right middle temporal lobe (T4, r = −0.098, *P* = 0.240, R^2^ = 0.0981) did not reach the significant level. In contrast, MMSE score was negatively correlated with only one brain region (O2 in right occipital lobe, r = −0.230, *P* = 0.047, R^2^ = 0.0529), and no clear correlation was found in other brain regions. The overall trend of the MMSE score was observed. R-squared values were also generally small (all R^2^ < 0.053). This suggests that the explanatory power of MMSE scores for the wavelength variation is very limited despite the negative association (Fig 6). Detailed statistical results are provided in S2 Table.

**Table 4:** Comparison of correlations between wavelength and cognitive scores (MoCA and MMSE) across brain regions.

Lead	MoCA (r)	MoCA (*P*)	MMSE (r)	MMSE (*P*)	Trend Consistency
Fp1	−0.371	0.003*	−0.175	0.102	MoCA significant
Fp2	−0.323	0.009*	−0.161	0.122	MoCA significant
F3	−0.270	0.024*	−0.114	0.207	MoCA significant
F4	−0.272	0.023*	−0.147	0.122	MoCA significant
C3	−0.330	0.007*	−0.165	0.117	MoCA significant
**C4**	**−0.207**	**0.067**	**−0.049**	**0.365**	**Both non-significant**
P3	−0.380	0.002*	−0.171	0.109	MoCA significant
P4	−0.257	0.030*	−0.093	0.251	MoCA significant
O1	−0.375	0.003*	−0.148	0.144	MoCA significant
**O2**	**−0.406**	0.001*	**−0.230**	0.047*	**Both significant**
F7	−0.321	0.009*	−0.160	0.124	MoCA significant
F8	−0.269	0.025*	−0.125	0.183	MoCA significant
T3	−0.335	0.007*	−0.165	0.117	MoCA significant
**T4**	**−0.098**	**0.240**	**−0.011**	**0.467**	**Both non-significant**
T5	−0.354	0.004*	−0.163	0.119	MoCA significant
T6	−0.339	0.006*	−0.208	0.066	MoCA significant

As shown in Fig 6A and 6B, the data points of MMSE score and wavelength were scattered and the regression line was flat, which intuitively reflected the weak correlation. The data points of MoCA score and wavelength in Fig 6C and 6D were closely distributed along the downward trend line, clearly and intuitively showing a stronger negative correlation trend.

## 4. Discussion

EEG activity recorded under resting-state, eyes-closed conditions is extensively employed in both clinical evaluations and academic investigations of diverse cognitive dysfunctions, attributed to its operational convenience and rich informational yield. Benz et al [30] conducted a comparative analysis of quantitative electroencephalography profiles in patients with MCI secondary to AD versus those with PD-MCI. They observed that PD-MCI patients exhibited a more pronounced slowing of the median EEG frequency relative to their AD-MCI counterparts. Furthermore, comparative studies between patients with AD and PDD have consistently indicated that EEG slowing is generally more severe in PDD [31,32]. These quantitative EEG signal feature differences may be related to the pathological synchronization of the sensorimotor-related slow-frequency brain motor system, which may be the cortical origin of the EEG in PDD.

Quantitative electroencephalography serves as a vital tool for identifying early biomarkers of neurodegenerative diseases and aids in the early detection and prognosis of dementia in studies on cognitive evolution in PD [33]. By introducing the phase average wavelength at the ninth scale as a new indicator, this study further elucidated the complex association between cognitive function and brain electrical activity in PD. The results demonstrate that, the wavelength of the whole brain in PD patients was significantly longer than that in normal controls. In the overall comparison of the ninth scale, the wavelength values of PD patients with cognitive impairment were generally larger than those of PD patients with normal cognition. When the analysis dimension was refined to each independent brain region, there was no significant difference between the PD patients with cognitive impairment group and the PD patients with normal cognition group in the phase average wavelength. Numerically, the PD patients usually showed longer wavelengths in frontal, central and anterior temporal regions. This phenomenon may stem from multifaceted neurophysiological and methodological reasons.

The whole brain wavelength of PD patients with cognitive impairment group was diffuse prolonged, which confirmed the correlation between cognitive impairment and overall slowing of brain electrical activity in PD [34,35]. The wavelength prolongation of frontal lobe, middle brain region and anterior temporal region is more significant, which may be closely related to the core role of these brain regions in cognitive function. Frontal lobe is the key brain region for executive function and attention regulation, and anterior temporal region is involved in memory encoding and semantic processing [36–39]. The dysfunction of these brain regions has been confirmed to be directly related to cognitive impairment in PD patients. The results suggest that the cognitive impairment of PD patients may be the disorder of electrophysiological rhythm with the focus on the core cognitive-related brain areas and the wide involvement of the whole brain. Its wavelength prolongation may reflect a decrease in the efficiency of information transmission in these brain intervals. The pathological processes of PD, such as dopaminergic neurons pathological degeneration and non-dopaminergic transmitter system disorders, will affect basal ganglia thalamo-cortical loops and change the synchrony and rhythmicity of neural oscillations [35,37,40–44].

Changes in neural oscillations in patients with PD may be caused by the disease itself and changes in cognitive status. Mild cognitive impairment in PD can occur in the early stage of the disease, often without obvious clinical cognitive symptoms or related complaints. Even if the patients themselves are not aware of subjective discomfort such as “deterioration of memory” and “slow brain”, some patients actually have objective cognitive decline [2,42,45–48]. The PD with normal cognition group and the PD with cognitive impairment group may share this underlying, broad background of electrophysiological abnormalities.

In addition, methodological factors cannot be ignored. Sample size, disease heterogeneity, and assessment tools may affect statistical power. The sample size of the PD with cognitive impairment group in this study was relatively small. At the same time, most of the MMSE scores in this group were concentrated in the high score range, which may dilute the differences between the groups. The cognitive screening tools used in this study, such as the MMSE, may not be sensitive enough to the mild cognitive impairment characteristic of PD, especially the problems in executive function, so that the grouping does not fully reflect the actual degree of neural network dysfunction.

Previous studies have established that the frequency of background rhythmic activity in EEG signals progressively decreases with the deterioration of cognitive function [30,49,50]. Considering the sensitivity of MMSE scale for cognitive function assessment [51–53]. The present study analyzed the correlation between this PAW metric and MMSE and MOCA scores. Results of this study showed a significant negative correlation between MoCA score and wavelength in most brain regions. Except for the O2 region, the correlation between MMSE score and wavelength did not reach statistical significance. R-squared values were also generally small, although MMSE score and wavelength showed a negative correlation. This difference is most likely due to the different cognitive dimensions assessed by the two scales and the differences in the distribution of cognitive states in the sample.

The number of PD patients with cognitive impairment was not large. There were few individuals with low MMSE scores (indicating moderate to severe impairment) in the sample. This distribution feature may weaken the correlation between MMSE and the phase average wavelength in two ways. On the one hand, the MMSE itself may not be very good at capturing cognitive changes in the early stages of PD, especially in executive functioning [54,55]. This means that some patients, who actually already have mild cognitive impairment, may still get a high score that is close to normal. This leads to the fact that people with actual differences in cognitive level are not reflected in MMSE scores, blurring the relationship between cognitive changes and EEG indicators. On the other hand, because MoCA is designed to be more sensitive to mild cognitive impairment, it can identify those with high MMSE scores who have already started to have mild cognitive decline [56–59]. Naturally, in the current sample, which was dominated by mild impairment, MoCA was able to map the cognitive decline more clearly, and thus show a stronger statistical relationship with brain wavelength.

The present study also found that the negative correlation between wavelength and MoCA score was more pronounced in the left hemisphere. In the right central area (C4) and middle temporal lobe (T4), the association was not significant. Existing imaging studies have told us that the left temporoparietal cortex of patients with early cognitive impairment in PD often shows metabolic reduction and atrophy earlier [60–63]. From the perspective of the test itself, the tasks such as verbal memory and fluency in the MoCA scale mainly rely on the network dominated by the left hemisphere [61,64]. The strong association of wavelength with the total MoCA score largely reflects its relationship with the cognitive network in the left hemisphere. This could also explain why wavelengths were less strongly associated with areas of the right hemisphere that are primarily responsible for sensorimotor or nonverbal memory, such as C4 and T4. PD patients had attenuated ~4 Hz EEG oscillatory activity at midfrontal electrodes in response to the interval-onset cue, which was correlated with MoCA scores [65]. These studies have shown that specific EEG features are associated with cognitive status in PD. Future studies with larger sample size and specific cognitive domains are needed to further explore the relationship between these areas and function.

## Conclusions

There was a broad negative correlation between the phase average wavelength of the alpha rhythm in PD patients and MoCA scores, and the strength of the correlation was significantly better than with MMSE. In other words, with the decline of cognitive function, the rhythm of alpha band slowed down. The analysis found that the phase average wavelength of scale nine in PD patients with normal cognition also changed, which may indicate a dual effect of cognitive impairment and PD pathology on brain function. Through the quantitative analysis of the phase average wavelength of alpha rhythm, this study indirectly uncovers potential alterations in the background alpha rhythm frequency of EEG signals. However, the relationship between this quantitative analysis index and various cognitive domains of cognitive function requires further investigation.

## Supporting information

S1 Table Results of pairwise statistical analysis on phase-averaged wavelength for the ninth scale. (DOCX)

S2 Table Proportion (R²) of Cognitive Scores (MMSE and MoCA) in explaining the variation of mean wavelength in each brain region. (DOCX)

S3 File Raw data. This file contains the complete anonymized raw dataset collected by the authors for this study. (XLSX)
