# Supplementary material for: The phase average wavelength of alpha rhythm in EEG signals of patients with Parkinson’s disease combined with cognitive impairment
Source: PLoS One. 2026 Apr 22;21(4):e0344786. doi: 10.1371/journal.pone.0344786 (PMC13102212; doi:10.1371/journal.pone.0344786)
Supplement: S1 Table — (DOCX) [file pone.0344786.s001.docx]

S1Table. Results of pairwise statistical analysis on phase-averaged wavelength for the ninth scale.

| Lead | Wavelength | | |
| --- | --- | --- | --- |
|  | NC vs PD-NC  (*P*) | NC vs PD-CI  (*P*) | PD-NC vs PD-CI  t(*P*) |
| FP1 | 0.000 | 0.000 | 0.223 |
| FP2 | 0.002 | 0.000 | 0.365 |
| F3 | 0.000 | 0.000 | 0.620 |
| F4 | 0.002 | 0.000 | 0.504 |
| C3 | 0.000 | 0.000 | 0.548 |
| C4 | 0.001 | 0.000 | 0.707 |
| P3 | 0.000 | 0.000 | 0.472 |
| P4 | 0.000 | 0.000 | 0.440 |
| O1 | 0.000 | 0.000 | 0.566 |
| O2 | 0.003 | 0.000 | 0.096 |
| F7 | 0.000 | 0.000 | 0.337 |
| F8 | 0.005 | 0.000 | 0.503 |
| T3 | 0.000 | 0.000 | 0.690 |
| T4 | 0.020 | 0.001 | 0.257 |
| T5 | 0.001 | 0.000 | 0.453 |
| T6 | 0.001 | 0.000 | 0.137 |

**Abbreviations**: PD-NC, PD with normal cognition; PD-CI, PD with cognitive impairment; NC, normal control group.
