# Supplementary material for: The phase average wavelength of alpha rhythm in EEG signals of patients with Parkinson’s disease combined with cognitive impairment
Source: PLoS One. 2026 Apr 22;21(4):e0344786. doi: 10.1371/journal.pone.0344786 (PMC13102212; doi:10.1371/journal.pone.0344786)
Supplement: S2 Table — (DOCX) [file pone.0344786.s002.docx]

**S2 Table. Proportion (R²) of Cognitive Scores (MMSE and MoCA) in explaining the variation of mean wavelength in each brain region.**

| Lead | MMSE (R²) | MoCA (R²) |
| --- | --- | --- |
| Fp1 | 0.0308 | 0.1373 |
| Fp2 | 0.0259 | 0.1046 |
| F3 | 0.0129 | 0.0726 |
| F4 | 0.0216 | 0.0738 |
| C3 | 0.0272 | 0.1086 |
| C4 | 0.0024 | 0.0428 |
| P3 | 0.0291 | 0.1447 |
| P4 | 0.0087 | 0.0660 |
| O1 | 0.0218 | 0.1408 |
| **O2** | **0.0529** | **0.1646** |
| F7 | 0.0255 | 0.1027 |
| F8 | 0.0157 | 0.0723 |
| T3 | 0.0272 | 0.1125 |
| T4 | 0.0096 | 0.0098 |
| T5 | 0.0267 | 0.1251 |
| T6 | 0.0431 | 0.1149 |
